# Evaluation of Awareness and Attitude of Telemedicine among Primary Healthcare Workers in Deprived Area Health Centers

**DOI:** 10.1155/2023/5572286

**Published:** 2023-09-26

**Authors:** Mahdi Mazandarani, Narges Lashkarbolouk, Mitra Hashemi

**Affiliations:** ^1^Endocrinology and Metabolism Research Center, Endocrinology and Metabolism Clinical Sciences Institute, Tehran University of Medical Sciences, Tehran, Iran; ^2^Golestan University of Medical Sciences, Gorgan, Iran; ^3^Deputy of Research and Technology, North Khorasan University Of Medical Sciences, Bojnurd, Iran

## Abstract

**Background:**

Telemedicine has the potential to make healthcare more efficient, organized, and available and is a more beneficial technology that can ease preventive treatment and improve long-term health management. This is especially essential for those who face financial or regional reasons to get quality treatment. Telemedicine in Iran is a new medical field and a noble way to access medical facilities for populations living in deprived areas, and the primary healthcare workers in these deprived medical centers are the implementers of telemedicine in those areas; we aimed to investigate the awareness and attitude towards telemedicine among all the healthcare workers in these centers.

**Method:**

This is a descriptive cross-sectional study at the Health Centers of Raz County in North Khorasan Province, Iran, and 149 healthcare workers were included. For collecting information, we used a questionnaire that consisted of two parts. The first part contains the demographic data of health care workers, and the second part includes the 5-point Likert scale questionnaire (questions on telemedicine awareness, attitude, and self-report readiness).

**Result:**

Most participants (51%) were male, and 69.8% were married. The most frequent sources of information about telemedicine are colleagues (40.3%), continuing education (24.7%), and social media and the internet (10.1%). Awareness did not significantly relate to gender, age, marital status, or work experience, but awareness of physicians and midwives is higher than other groups (*p* < 0.05). The awareness of healthcare workers using continuing education, articles, workshops, or conferences was significantly higher (*p* < 0.05). The attitude scores for most questions are above 3.4 and reflect a positive attitude about telemedicine. Attitudes did not show a significant relation to gender, age, marital status, or work experience.

**Conclusion:**

Using telemedicine in developing countries, rural or urban areas have a high potential to improve epidemiological investigations, disease control, and clinical case management. Providing healthcare professionals with more information about new technologies in healthcare, such as telemedicine, can help get a more realistic picture of their perceptions.

## 1. Introduction

Telemedicine is a health-related service using telecommunications and electronic information technology. Over the past decade, advanced technologies with quality network services have enabled individuals to improve healthcare delivery and make it accessible to more patients. It is an essential and beneficial technology that can ease preventive treatment and improve long-term health. In addition, it can make healthcare more efficient, organized, and available. This is increasingly being adopted to bring specialty-palliative care into the homes of seriously ill patients, improve chronic disease management, aid in emergency medicine, and as a critically essential service during pandemics [1–5].

Current trends in medical education observe the development of information and communication technologies, where mobile technology and health applications have shown great potential to influence the timely diagnosis and treatment of diseases and aid in diagnostic evaluations at the clinical level. Due to the increase in the elderly population, the decrease in visits to health and medical centers due to the COVID-19 pandemic, and the lack of specialized health-work staff in the deprived and border areas of the country, it is necessary to produce new, accurate, and affordable methods to help physicians. Telemedicine has improved healthcare providers' capacity to care for many patients without physical presence. It has many advantages, including online consultation, remote monitoring, remote nursing, and physical and psychiatric rehabilitation. It enables better healthcare choices, increases the quality and efficiency of emergency services, reduces the time needed to establish a diagnosis, and reduces financial health-related burdens by optimizing streamlined clinical processes and reducing hospital travel costs [3, 4, 6–12].

Telemedicine will be conducted by text, audio, and video, allowing an individual risk assessment to determine if the patient has an appropriate case definition. Studies stated that patients who receive care by telemedicine are typically satisfied with the convenience, efficacy, and time-saving. With the experience of the COVID-19 pandemic, telemedicine has become a vital service for patients to help mitigate and control the spread of epidemics and increase personal protection in addition to meeting the medical needs of patients. Research in this area is still preliminary, but it is expanding. This is especially true for those who face financial or regional challenges to get quality treatment. In Iran, most telemedicine research has been conducted on healthcare workers in referral hospitals, and the awareness and attitude of telemedicine in rural and border medical centers, which are the main goals of its establishment, have yet to be studied [13–17].

Considering that telemedicine in Iran is a new medical field and a noble way to access medical facilities for populations living in deprived areas is one of its goals. Moreover, the healthcare workers in these deprived medical centers are the implementers of telemedicine in those areas; we conducted this study to investigate the awareness and attitude towards telemedicine among all the healthcare workers in these centers.

## 2. Methods

### 2.1. Study Design and Participants' Selection

This is a descriptive cross-sectional study at the Health Centers of Raz County, Bojnord, Iran. The study population included physicians, nurses, midwives, health personnel, and laboratory and radiological technicians. The sampling method in this study was a census, and all health network staff members who met the entry criteria were invited to participate. Out of 162 healthcare staff, 149 (91.9%) ultimately completed the study. The study's inclusion criteria are all members of the Health Centers of Raz County and treatment network workers. The exclusion criteria were lack of cooperation of health care workers and failure to complete the questionnaire.

### 2.2. Data Collection

For collecting information, we used a questionnaire that consisted of two parts. The first part contains the demographic data of health care workers (age-gender-marriage-education-work experience), and the second part includes the 5-point Likert scale questionnaire (questions on telemedicine awareness (8 questions), attitude (19 questions), and self-report readiness (3 questions)).

A questionnaire with a 5-point Likert scale was implemented to collect information on telemedicine awareness, attitudes, and self-reported readiness. The questions were established based on a review of existing literature. To ensure the validity of the questionnaire, feedback was acquired from five specialists in medical and clinical informatics and health information. They assessed the format, clarity, and meaning of the questions and response options. The reliability of the questionnaire was investigated by having 30 staff members complete it and calculating Cronbach's alpha, which resulted in a value of 0.85 [13].

Scoring of awareness based on the Likert scale was assigned as very little (1 point), little (2 points), some (3 points), enough (4 points), and very much (5 points). Attitude scores were to disagree (1 point) completely, disagree (2 points), agree somewhat (3 points), agree (4 points), and completely agree (5 points). In this study, a questionnaire was used based on the analysis of Sheikhtaheri et al. in 2016, conducted on Mashhad Hospital employees [13].

### 2.3. Statistical Analysis

The Statistical Package for Social Sciences (SPSS) for Windows version 20.0 (SPSS, Chicago, IL) was used for all statistical analyses. Continuous variables were expressed as mean ± standard deviation (SD), and median and categorical variables were expressed as counts (percentage). The data is analyzed using SPSS-20 software using the Mann–Whitney, Kruskal-Wallis, and Spearman correlation tests. Descriptive statistics indicators of mean, median, and standard deviation will describe data from tables and graphs. A *p* value less than 0.05 was considered significant.

### 2.4. Ethics Approval

Informed consent was obtained from all individual participants, and a copy of the written consent is available for review by the editor of this journal. The purpose of this study was completely explained to the participants, who were assured that all the information would be kept confidential by the authors. This study was conducted in line with the principles of the Declarations of Helsinki. The ethical committee of North Khorasan University of Medical Sciences approved (approved ethic number: IR.NKUMS.REC.1401.089).

## 3. Result

Most participants (51%) were male, and 69.8% were married. Age ranged from 22 to 61 years old, and the mean age was 33.73 ± 7.74 years old. The most common educational level of the participants (57%) was a bachelor's degree, and the mean occupational experience was 5.81 ± 5.04 years old.

One hundred forty-three had no experience with telemedicine programs, and six had telemedicine experience. The most frequent sources of information about telemedicine are colleagues (40.3%), continuing education (24.7%), and social media and the internet (10.1%). Other sources include articles (9.2%), workshops (7.8%), formal education (5.5%), and conferences (2.4%).

Awareness of telemedicine in healthcare workers is low (2.6 out of 5), and it did not significantly relate to gender, age, marital status, or work experience, but awareness of physicians and midwives is higher than other groups (*p* < 0.05). The awareness of healthcare workers using continuing education, articles, workshops, or conferences was significantly higher (*p* < 0.05) (Table 1).

The Mann–Whitney test did not show a significant difference between the average scores of attitude and awareness based on marital status and gender (*p* = 0.16 and *p* = 0.37 for marriage and *p* = 0.69 and *p* = 0.19 for gender).

Spearman's test did not indicate a significant relationship between work experience or age in the knowledge and attitude scores (*p* = 0.42 and *p* = 0.1 for work experience and *p* = 0.62 and *p* = 0.99 for age.)

The attitude scores for most questions are above 3.4 and reflect a positive attitude about telemedicine. Attitudes did not show a significant relation to gender, age, marital status, or work experience (Table 2). The more familiar employees are with computers and information technology, the more positive their attitudes towards telemedicine's impact on improving clinical decisions and follow-up and reducing medical errors, and they make a more positive assessment of their organization's readiness (*p* < 0.05).

## 4. Discussion

Telemedicine technologies have been touted as an elegant solution to the challenges of providing equitable, cost-effective, and efficient healthcare services for over two decades. This is especially true for those living in rural, remote, or urban areas, especially in developing countries, who have limited healthcare options or cannot afford other forms of health. The sensory aspects of healthcare and the physician-patient relationship mediated by telemedicine significantly contribute to success, failure, or unintended consequences [1–7, 9, 12–18].

In our study, the most common educational level of the participants (57%) was a bachelor's degree, and the most frequent sources of information about telemedicine are colleagues (40.3%), continuing education (24.7%), and social media and the internet (10.1%). In the study by Ghazi and Tanhapour in 2022, they stated that 57.3% of providing training and 17.4% of consulting were the best uses of telemedicine. Also, 60% of the specialists considered the lack of technical workers and 17.02% of primary costs as the biggest obstacles to using telemedicine in the hospital and assuming the existence of a favorable executive culture and the existing appropriate infrastructure and the current level of awareness among the specialists of this medical center. In our study, awareness of telemedicine among healthcare workers is low (2.6 out of 5). Awareness did not show a significant relation to gender, age, marital status, or work experience, but awareness of doctors and midwives is higher than other groups (*p* < 0.05). The awareness of healthcare workers using continuing education, articles, workshops, or conferences was significantly higher (*p* < 0.05). Telemedicine technology is a new method in Iran; it does not have a sufficient place in the educational curricula of medical sciences, and on the other hand, many primary healthcare staff have not completed courses related to it during their education. In addition, the lack of necessary infrastructure and internet network in these areas, along with the distance from the capitals of the provinces and the country, the lack of trained healthcare staff, and the absence of comprehensive guidelines have led to a low level of awareness among healthcare staff in primary healthcare centers. Furthermore, it should be added that the geographical locations and the restrictions for the employees of deprived health centers have helped to intensify these factors. A low level of awareness of the healthcare staff regarding telemedicine and its positive capabilities can lead to disruption in the implementation process and an increase in cost and time for its administration. This lack of awareness can also decrease its performance and active role even after implementation. Increasing training programs for healthcare staff in these areas and solving other fundamental obstacles (including financial support, the necessary infrastructure, and understanding the religious, ethnic, and cultural issues of the people in these areas) can be a suitable way to increase its efficiency in deprived areas. The attitude scores for most questions are above 3.4 and reflect a positive attitude about telemedicine. Attitudes did not show a significant relation to gender, age, marital status, or work experience [13, 16, 19–23].

The study by Sheikhtaheri et al. reported that the awareness score is 13 ± 5.5 out of 35, indicating low awareness of telemedicine. Only 43.7% answered that they had heard about telephone counseling. Awareness of other types of telemedicine services was even lower. The most commonly used sources of information about telemedicine were friends (51.4%) and public media (30.3%). Attitudes towards telemedicine were generally positive (63.42 ± 9.5 of 95). We found a significant positive correlation between attitudes and awareness (*p* = 0.027).

The experience and role of telemedicine during the COVID-19 epidemic and its national lockdown, which led to a decrease in the communication between patients and healthcare staff, are another example of the importance of telemedicine in today's modern medicine. During the epidemic, many patients continued to follow-up on their disease and treatment and continued communication through telemedicine systems. Various studies were also published in this regard, showing telemedicine's successful role in this period. In Iran, although telemedicine is considered a new method, patients have been able to follow-up and receive proper treatment through this method. This issue is especially vital for chronic patients who need continuous follow-up to maintain their health. The study of Chunara R.,et al.2021, intended to investigate the effect of telemedicine during the COVID-19 pandemic on 8077 patients, and they stated that during the pandemic and lockdown, the importance and use of telemedicine in young, female, and Black population have increased, and the effect is very high. Data from patients' electronic health records were used to perform descriptive and multilevel analyses according to the type of visit (remote or face-to-face medicine) and suspected COVID-19. Controlling for individual and community-level characteristics, Black patients had 0.6 times the adjusted odds (95% CI: 0.58–0.63) of accessing telemedicine care compared to White patients. Although telemedicine has not solved all the challenges and problems caused by the pandemic and home lockdown, it has improved care and medical services in America [7, 8, 10–16, 18–26].

Moreover, during the COVID-19 pandemic, numerous hospitals were dedicated to treating COVID-19 patients, and fewer hospital beds were available to other patients, so telemedicine became more critical. This issue led to an increased need for interdisciplinary cooperation. During this period, many specialists were required to use telemedicine and multidisciplinary collaboration to treat patients. These new collaborations increased the quality of medical services to patients, especially those in rural areas who needed instant or continuous follow-ups. This cooperation would not have been possible without the availability of telemedicine technologies [27].

Several studies have been conducted on the obstacles to establishing and implementing telemedicine in developing countries. According to the systematic review study conducted by Al-Samarraie H.,et al.2020, the barriers in developing countries in the Middle East were divided into six categories. These include (1) cultural (religious and social constraints, resistance to change, traditional beliefs, literacy levels, and language differences), (2) financial (lack of financial support, feasibility studies, and satisfaction with telemedicine technology), (3) organizational (existing infrastructure, professional expertise, strategic planning, training, effective monitoring, media representation, insurance and reimbursement problems, insufficient ICT (information and communication technologies), and broadband Internet connection in rural areas), (4) legal and regulatory (security, safety, confidentiality for data protection, and ethical issues), (5) individual (lack of experience, awareness, knowledge, trust, motivation, and satisfaction in using technology), and (6) technological (lack of system quality and technical support) [28].

One of the positive results of telemedicine is reducing costs related to transporting patients and increasing the speed of making decisions and treating patients. In our study, 73.9%, 92.7%, and 92.6% of participants had a positive attitude towards improving clinical decisions, clinical workflow, and an increase in the speed of medical services. In line with our result, a study by Atmojo JT.,et al.2020, found that telemedicine can reduce patient transportation costs and speed treatment decision-making. In addition, it ensures patient satisfaction across a range of parameters, including improved medical outcomes, ease of use, improving patient satisfaction through improved communication, reduced travel time to the hospital, improved access, and reduced readmissions [5, 6, 11, 12, 19–24].

There are no specific courses or guidelines on this technology for clinical health workers in Iran. Graduates of clinical fields have not received extensive training in this regard. The results show that the most positive attitude is related to quick information access and improving information quality. Better access to patient records can improve document quality and timeliness of information. Physicians believe that telemedicine will be useful if it improves patient care, documentation, and timeliness and enables low-cost and high-accuracy patient monitoring [23–26, 29–33].

Limited information about telemedicine has affected clinicians' perception of this technology. Therefore, before implementing this technology, it is essential to increase users' knowledge about it and demonstrate its capabilities and benefits. Sufficient knowledge and a positive perception of technology are the main factors that encourage users to use technology in the future [24, 26, 29–33].

In all countries, most of the research has been done on healthcare workers and physicians in hospitals, and the importance of telemedicine awareness in rural health centers and borders, which are the primary targets of telemedicine, has yet to be studied. In our study, the awareness and attitudes of health center workers in border and developing areas were investigated, which is one of the strengths of our research. We suggest that the development of guidelines and improvement in the information technology (IT) skills of clinical health workers could increase readiness to accept telemedicine. The limitation of our study is that patients' attitude and satisfaction was not investigated, and there is not enough information in this regard.

## 5. Conclusion

In recent years, the role of telemedicine in postoperative care has attracted attention as it has demonstrated excellent clinical outcomes, increased patient satisfaction, increased accessibility, reduced waiting times, and saved costs for patients and the health system. Providing healthcare professionals with more information about new technologies in healthcare, such as telemedicine, can help get a more realistic picture of their perceptions.

## Figures and Tables

**Table 1:** Clinical healthcare workers' awareness about telemedicine.

Awareness of	Very little *N* (%)	A little *N* (%)	Some *N* (%)	Enough *N* (%)	Very much *N* (%)	Mean ± SD
Overview of telemedicine	33 (22.1%)	0 (0%)	17 (11.4%)	67 (45%)	32 (21.5%)	2.65 ± 1.05
Effect of telemedicine on practice	1 (0.7%)	54 (36.2%)	62 (41.6%)	32 (21.5%)	0 (0%)	2.83 ± 0.76
Effect of telemedicine on income	12 (8.1%)	80 (53.7%)	57 (38.3%)	0 (0%)	0 (0%)	2.3 ± 0.61
Effect of telemedicine on quality	18 (12.1%)	37 (24.8%)	0 (0%)	62 (41.6%)	32 (21.5%)	2.72 ± 0.93
Effect of telemedicine on the number and staff members needed	29 (19.5%)	25 (16.8%)	73 (49%)	0 (0%)	22 (14.8%)	2.73 ± 1.21
Effect of telemedicine on patient education	29 (19.5%)	25 (16.8%)	63 (42.3%)	32 (21.5%)	0 (0%)	2.65 ± 1.02
Telemedicine infrastructure	23 (15.4%)	96 (64.4%)	2 (1.3%)	28 (18.8%)	0 (0%)	2.23 ± 0.93

SD: standard deviation.

**Table 2:** Clinical healthcare worker attitude about telemedicine.

I believe that telemedicine may…	Completely disagree *N* (%)	Disagree *N* (%)	Agree somewhat *N* (%)	Agree *N* (%)	Completely agree *N* (%)	Mean ± SD
Reduce medical errors	2 (1.3%)	11 (7.3%)	27 (18%)	88 (58.6%)	22 (14.6%)	3.78 ± 0.42
Facilitate job duties	6 (4%)	12 (8%)	15 (10%)	90 (60%)	27 (18%)	3.75 ± 0.59
Increase communication among providers	10 (6.6%)	20 (13.3%)	40 (26.6%)	23 (15.4%)	57 (38%)	4.04 ± 0.92
Increase information for patients	0 (0%)	28 (18.8%)	32 (21.5%)	23 (15.4%)	66 (44.3%)	3.85 ± 1.18
Increase the speed service	0 (0%)	0 (0%)	11 (7.4%)	18 (12.1%)	120 (80.5%)	4.73 ± 0.59
Increase timely access to information	0 (0%)	0 (0%)	0 (0%)	50 (33.6%)	99 (66.4%)	4.67 ± 0.47
Improve quality of healthcare information	0 (0%)	0 (0%)	22 (14.8%)	56 (37.6%)	71 (47.7%)	4.32 ± 0.72
Improve clinical decisions	0 (0%)	17 (11.4%)	22 (14.8%)	39 (26.2%)	71 (47.7%)	4.1 ± 1.08
Improve clinical work flow	0 (0%)	11 (7.4%)	0 (0%)	67 (45%)	71 (47.7%)	4.32 ± 0.81
Provide more comprehensive healthcare services	0 (0%)	0 (0%)	11 (7.4%9	99 (66.4%)	39 (26.2%)	4.18 ± 0.54
Improve tracking of patient health status	0 (0%)	0 (0%)	17 (11.4%)	93 (62.4%)	39 (26.2%)	4.14 ± 0.6
Threaten staff positions	39 (26.2%)	77 (51.7%)	0 (0%)	22 (14.8%)	11 (7.4%)	2.25 ± 1.2
Reveal weak points	0 (0%)	39 (26.2%)	39 (26.2%)	43 (28.9%)	28 (18.8%)	3.4 ± 1.07
Increase staff work load	0 (0%)	0 (0%)	0 (0%)	11 (7.4%)	138 (92.6%)	4.9 ± 0.26
Create new responsibilities for staff	0 (0%)	20 (13.3%)	0 (0%)	56 (37.6%)	73 (48.6%)	4.62 ± 0.48
Threaten information confidentiality	72 (48.3%)	11 (7.4%)	6 (4%)	58 (38.9%)	2 (1.3%)	2.37 ± 1.44
Threatening patient privacy	67 (45%)	0 (0%)	0 (0%)	71 (47.7%)	11 (7.4%)	2.72 ± 1.58
Increase costs	50 (33.6%)	27 (18.1%)	33 (22.1%)	22 (14.8%)	17 (11.4%)	2.52 ± 1.38
Increase legal challenges	17 (11.4%)	0 (0%)	39 (26.2%)	39 (26.2%)	54 (36.2%)	3.7 ± 1.26

SD: standard deviation.

## Data Availability

Data and material of this article are not publicly available due to ethical matters but are available from the corresponding author on reasonable requests.
